# A systematic review of the relationships between social capital and socioeconomic inequalities in health: a contribution to understanding the psychosocial pathway of health inequalities

**DOI:** 10.1186/1475-9276-12-54

**Published:** 2013-07-19

**Authors:** Eleonora P Uphoff, Kate E Pickett, Baltica Cabieses, Neil Small, John Wright

**Affiliations:** 1 Department of Health Sciences, University of York, Seebohm Rowntree Building, York YO10 5DD, UK; 2 Faculty of Medicine Universidad del Desarrollo, Santiago, Chile; 3 School of Health Studies, University of Bradford, Richmond Road, Bradford BD7 1DP, UK; 4 Bradford Institute for Health Research, Bradford Royal Infirmary, Duckworth Lane, Bradford BD9 6RJ, UK

## Abstract

**Introduction:**

Recent research on health inequalities moves beyond illustrating the importance of psychosocial factors for health to a more in-depth study of the specific psychosocial pathways involved. Social capital is a concept that captures both a buffer function of the social environment on health, as well as potential negative effects arising from social inequality and exclusion. This systematic review assesses the current evidence, and identifies gaps in knowledge, on the associations and interactions between social capital and socioeconomic inequalities in health.

**Methods:**

Through this systematic review we identified studies on the interactions between social capital and socioeconomic inequalities in health published before July 2012.

**Results:**

The literature search resulted in 618 studies after removal of duplicates, of which 60 studies were eligible for analysis. Self-reported measures of health were most frequently used, together with different bonding, bridging and linking components of social capital. A large majority, 56 studies, confirmed a correlation between social capital and socioeconomic inequalities in health. Twelve studies reported that social capital might buffer negative health effects of low socioeconomic status and five studies concluded that social capital has a stronger positive effect on health for people with a lower socioeconomic status.

**Conclusions:**

There is evidence for both a buffer effect and a dependency effect of social capital on socioeconomic inequalities in health, although the studies that assess these interactions are limited in number. More evidence is needed, as identified hypotheses have implications for community action and for action on the structural causes of social inequalities.

## Introduction

Since the late 1980’s, the concept of social capital has gained prominence in research and in the discourse of policy making [1]. This prominence has emerged from, and built upon, a long tradition in sociology and interdisciplinary fields of study that have considered patterns in human relationships and links with social solidarities. In the field of health inequalities research the social environment is acknowledged as a multi-faceted social determinant of health that can promote or harm health through multiple mechanisms [2]. There is an abundance of evidence that confirms the relationship between different measures of social capital and health, and some evidence that social capital mediates the relationship between income inequality and health [3]. However, the relationship between social capital and socioeconomic inequalities in health remains unclear. We aim to clarify this relationship, as knowledge of the pathways involved in the development and maintenance of health inequalities can inform changes that contribute to a healthier society for all.

### Defining and measuring social capital

Despite its potential to clarify the origin of health inequalities, the use of social capital has suffered from a lack of consensus regarding its definition and measurement [4,5]. Sociologists [6,7], economists and political scientists have made major contributions to the theoretical framework [8,9]. As a comprehensive theoretical overview goes beyond the purpose of this paper, we refer interested readers to key texts by Coleman [7], Bourdieu [6] and Putnam [9]. Putnam regards social capital above all as an attribute of society, and its value lies in social networks and the norms of reciprocity and trustworthiness that arise from them [9,10]. Changes in social capital over time are attributed to structural societal changes instead of individual influences [9]. Bourdieu emphasises the way that social capital reproduces inequality by allowing some people to mobilise the capital of their family, sports club, school or other associations to their advantage. He defines social capital as: ‘the aggregate of the actual or potential resources which are linked to possession of a durable network of more or less institutionalized relationships of mutual acquaintance or recognition’ [6]. Instead of Putnam’s idea of social capital as a freely available community resource, Bourdieu argues that a lack of economic and cultural capital creates barriers for subgroups in society to acquire and use social capital. Coleman [7] approached social capital as a way of integrating social theory with economic theory using “rational action theory”. He argued that social capital involves an expectation of reciprocity within networks characterised by high degrees of trust and shared values. According to Coleman social capital constitutes a public good, benefiting all those who are part of a structure and, as such, it is a potential asset for the underprivileged and not just an instrument of privilege.

In order to measure and utilise social capital in research, the concept is often deconstructed into bonding, bridging and linking components [11,12]. Bonding social capital refers to close relationships between family members or good friends, measured by indicators such as social support. These relations form a strongly tied network based on a shared social identity. Bridging social capital is based on Granovetter’s idea of ‘weak ties’ [13] and refers to relationships between people who are more loosely connected and have a distinct social identity, such as neighbours, members of a sports club or colleagues [14]. Linking social capital is used to describe relationships that are characterized by power differences, such as the hierarchical relationship between employer and employee, or between citizen and government.

A distinction can also be made between structural and cognitive components of social capital [12]. Cognitive social capital refers to the social cohesion keeping networks together, measured by subjective indicators such as trust, social support and neighbourhood satisfaction. Structural social capital refers to objectively measurable activities and resources such as participation in neighbourhood activities, membership of a religious association or election turnout. It facilitates sharing of knowledge and collective action.

### Social capital and health inequalities: the theory

In high-income countries, each step down the social ladder is associated with worse health outcomes [15]. This ‘social gradient’ suggests that social inequalities in health do not only reflect material disadvantage related to socio-economic status, but also a psychosocial pathway associated with social position [16]. Two mechanisms through which the psychosocial pathway operates are the limited availability and utility of social capital and the stress arising from status comparisons [17]. In this paper we focus on the role of social capital in the production of socioeconomic inequalities in health.

At the individual level, social capital can counteract the negative effects of stress or improve one’s ability to cope with stress by enhancing emotional or financial support [16]. A healthier way of coping with stress may mean people are less likely to smoke, consume alcohol or indulge in comfort eating as coping mechanisms [1].

Recently, research interests have shifted from assessing social capital at an individual level to applying an area-level focus often referred to as ‘contextual social capital’. At the community level, the influence of social networks and norms could have a health effect in addition to the effects of individual social capital. The social space, rather than the individuals who live in it, is the reservoir of social capital [18]. Examples of mechanisms related to social capital that operate at the community level are the presence of health-related social norms, collective efficacy facilitating collective action, reciprocity and diffusion of health-related information [19].

Societies with a higher level of social equality seem to enjoy higher stocks of social capital and have better health outcomes, together with a lower incidence of social problems such as violence, drug abuse, school drop-outs and teenage pregnancies [20]. Social capital creates solidarity, stimulating the government to opt for fairer policies aimed at reducing health and social inequalities [3]. Simultaneously, it might enhance the capacity of the socially privileged to further bolster their position. Both Bourdieu and Coleman argue that social capital might improve health but may also exacerbate inequalities. Not everyone has access to the same sources of social capital and not everyone will benefit in the same way.

When studying contextual social capital, the key is to distinguish between the effect of individual social resources on health and the health effects that can be attributed to characteristics of the wider environment. This contextual perspective poses measurement challenges. Individuals usually report on their own social support, level of trust, social participation or other indicators. While some structural measures, such as a count of voluntary organisations in an area, by-pass this problem, they provide very general indicators. These measures fail to address individual differences between the people that take part in activities or organisations, for example with regard to their socioeconomic status. This becomes problematic when contextual social capital has different effects on health and wellbeing for different individuals or groups in a community.

### Hypotheses of interaction

**Figure 1:**
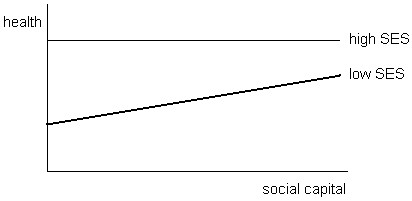
The buffer effect of social capital on socioeconomic inequalities in health.

Given the likelihood that the effects of social capital on health will vary in size and nature between groups with different positions in society, it is worthwhile considering the influence of social inequality. We will offer three main hypotheses on the interaction between social capital, socioeconomic inequalities and health. Firstly, components of social capital such as social cohesiveness can provide a *buffer* against stress and other negative influences on health and wellbeing in tight-knit communities. It has been suggested that in areas with a high density of ethnic minorities, the social network serves as a spatial barrier against the negative impacts of discrimination or stigmatization on health [21]. Although this ethnic density hypothesis has been explored independently of social capital theory, the protective health effects resulting from strong ethnic bonds show the importance of bonding social capital for health. Ethnic minority groups often occupy lower positions on the social ladder. Since their social disadvantage is based on a lack of power rather than numbers, they can be considered a minority in a neighbourhood where they constitute the majority of residents, as is the case with many Black Americans or Pakistani UK residents. Other studies provide examples of solidarity among working class communities being manifest in health initiatives, for example around chronic illnesses associated with particular industries [22].The buffer hypothesis suggests a greater benefit of social capital on health for people with a disadvantaged position in society, and no effects or limited health benefits for those with a position higher up the social ladder. People with high levels of social capital would be healthier than expected considering their low socioeconomic status. This effect is illustrated in Figure 1.

**Figure 2:**
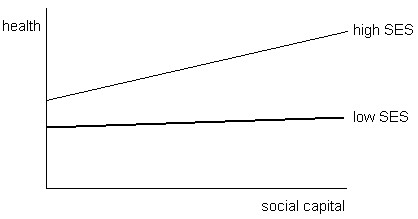
Dependency of social capital and socioeconomic satus influencing health.

A second hypothesis, based on Bourdieu’s [6] model of social capital, suggests a *dependency* between social, economic and cultural capital (Figure 2). Economic and cultural capital is required in order to use and accumulate social capital for the benefit of health. In health research, economic and cultural capital is often combined into measures of socioeconomic position. This review will consider the distinction between economic and cultural capital by developing a nuanced understanding of social capital as including aspects that foster both local area solidarities, bonding social capital, and those that can link individuals or groups with different levels of economic and cultural capital, bridging social capital. People with a low socioeconomic status will generally have less social capital and the amount of capital available to them cannot be used as effectively for the benefit of health. Although seemingly in contrast with the *buffer* hypothesis, these two ideas do not necessarily contradict each other. Socially disadvantaged individuals might benefit from bonding social capital in closely connected family or community networks, but miss out on the beneficial effects of bridging social capital.

**Figure 3:**
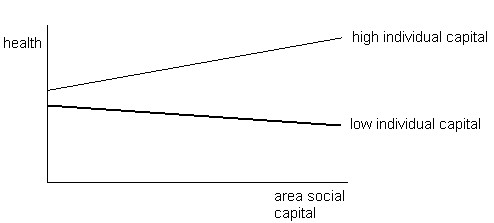
Effect of contextual social capital on socioeconomic inequalities in health.

The third hypothesis relates to the effect of *contextual* social capital on health, as opposed to social capital measured at an individual level that is considered to be an attribute of the individual. In line with the dependency hypothesis, it has been argued that social capital might not be available or beneficial to everyone living in an area. Mechanisms of control and social pressure can cause social exclusion [23]. Social capital might benefit the better-off in society, while excluding people with a lower socioeconomic status or minority position (Figure 3). For those lower on the social ladder, being surrounded by inaccessible social capital might lead to further deteriorations in health.

#### Research aim

This paper offers two contributions to the debate on social capital and inequalities in health. First, we offer a systematic review of published papers on this topic. As far as we are aware, Carlson and Chamberlain [24] have performed the only overview of social capital in relation to health inequalities. Although the authors discussed the implications of their findings for health disparities, they did not include any inequality-related terms in the search strategy. Their review included studies published from 1997 to 2002 and they used a restricted version of social capital, mainly focussing on the measure of civic trust while excluding concepts such as social cohesion. Their approach has captured only part of the body of work that has developed social capital conceptually and empirically. No overview has been presented on the different types of social capital, economic inequality and health outcomes used in research, and it is unknown which measures are most likely to show significant correlations [25].

Second we hope to find evidence for interaction effects between socioeconomic position and social capital in relation to health. Above we have introduced three hypotheses to help frame our findings. In pursuing this aim we move beyond a conventional systematic review in which current evidence and gaps in knowledge are identified and we offer an interpretation of the associations and the pathways between social capital and socioeconomic inequalities in health.

## Methods

The methods and results of this systematic review are reported according to the PRISMA guideline to facilitate the transparency and reproducibility of our findings [26]. The search strategy and selection of studies was deliberately broad to allow for a wide variety of study designs and interpretations of social capital to be included. We reviewed studies published before July 2012 that could be located through online databases MEDLINE, EMBASE, CINAHL and Cochrane. The search identified studies that included terms related to social capital, health inequalities and/or socioeconomic status in the article, title or abstract. The complete search in all four databases is documented in detail in Additional file 1.

Studies were included regardless of study design, setting, social capital measure, type of health outcome and date of publication. No language restrictions were applied. Grey literature was excluded and background papers and reviews were separated from the main results.

**Figure 4:**
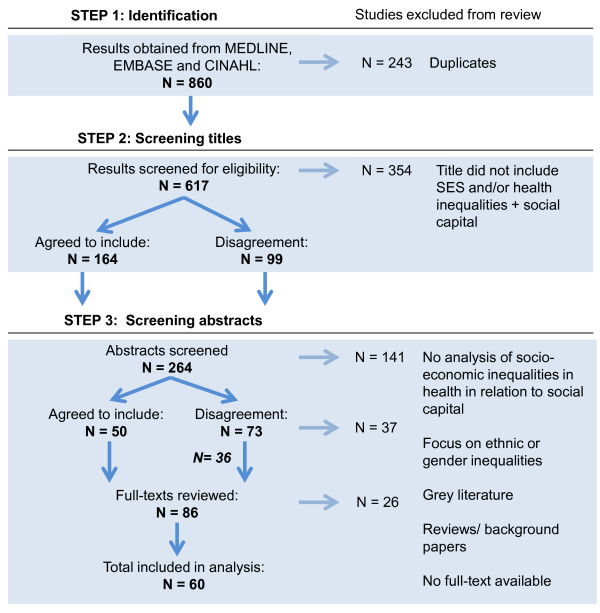
Selection process systematic literature search.

The systematic literature search was performed on the 25th of July 2012 in all four databases and step 1 resulted in the identification of 618 studies after removing duplicates (Figure 4). In step 2 titles were independently screened by NU and BC and in step 3 studies for which inclusion was agreed and studies on which no first agreement was reached were reviewed. Abstracts were assessed by two authors independently (NU and BC) and rejected if they did not analyze socioeconomic inequalities in health in relation to social capital or any of the related indicators. Abstracts of studies not agreed upon after this step were discussed until complete agreement between the two researchers was reached to either exclude or include the study for further analysis. A table with excluded studies after disagreement and reasons for exclusion can be found in Additional file 2.

The full-text review and data extraction were performed by one reviewer (NU) based on a summary table developed and piloted by the research team. (Additional file 3). A second reviewer (BC) checked a random 10% sample of the completed summary table. After the full-text review, studies were excluded if they failed to address social capital or any of the related indicators, if they did not use any health outcomes or if they did not include any measure on socioeconomic status or health inequalities. No summary measures were reproduced given the incomparability of dependent and independent variables used in the studies.

### Quality assessment

Given that most of the criteria for risk of bias provided by the PRISMA statement are related to trials with a more biomedical orientation, we assessed the quality of the study rather than the risk of bias. Firstly, the suitability of social capital and economic capital measures in relation to the aim or research question was assessed. This included an examination of potential logical fallacies and we verified whether a sound theoretical motivation for the choice of the social capital measure or related indicator was provided. Transparent use of the social capital concept was emphasized; we expected studies to either use measures independently of the social capital concept, for example ‘trust’, or to use measures such as social support as indicators of social capital. Secondly sample size and design of the study were assessed in relation to the type of analyses and reported conclusions. Studies were assigned one point if they failed on any of these quality criteria, two if there was substantial room for improvement and three if all quality criteria were met.

## Results

A total of 60 studies were included in the analysis. A summary table of selected studies and main results is presented in Additional file 3. The collected data represents an array of geographical regions, with studies from the United States, Europe, Asia, Australia, Canada and the former Soviet Republic. Studies relying on data from the United States made up the biggest portion, but due to smaller sample sizes these participants represent only 24% of the total sample.

Self-reported measures of health were most frequently reported, and used as the only measure in 42% of all studies. Other indicators of health and illness were measures of health behaviour, hypertension, obesity, mental health, mortality, access to care or a combination of multiple measures.

### Correlation between social capital and socioeconomic inequalities in health

**Figure 5:**
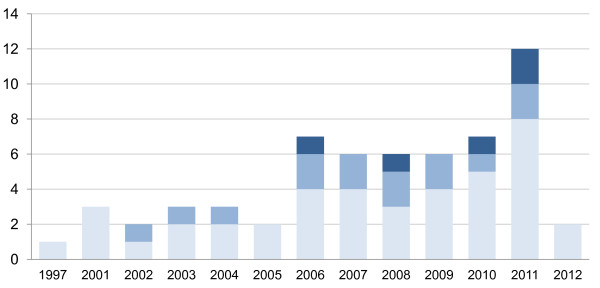
**Trend of research on interaction social capital and socioeconomic inequalities in health.** Light Blue rectangle, No interaction tested; Blue rectangle, Interaction confirmed; Dark Blue rectangle, No interaction found.

Figure 5 shows nineteen studies testing for interaction effects of social capital and socioeconomic inequalities in health. The remaining studies assessed the correlation between social capital, health and socioeconomic status without taking into account interaction effects.

Out of sixty studies reviewed for analysis, only four did not confirm this three-way correlation. One of the studies did not analyse this hypothesis [27], another study only used structural measures of social capital to test the relationship with self-rated health [28] and two studies did not find an effect of social capital on mortality [29,30]. Turrell and colleagues [29] attributed this finding mainly to a lack of spatial segregation within the study population of Tasmania, while Mohan and colleagues [30] focussed on area-level measures of social capital.

The studies that did confirm this hypothesis were mainly cross-sectional studies, often making use of data from large surveys. Sixteen studies analysed a sample consisting of more than 8000 people, representing countries with low levels of socioeconomic inequality ( e.g., Sweden and Norway), high-income countries with relatively high inequality (e.g., the United States and United Kingdom), and middle-income countries with high inequality (e.g., countries from the former Soviet Republic).

The studies revealing a relationship between social capital and socioeconomic inequalities in health often included multiple measures of social capital or related concepts, although the choice for these measures and components was not always clearly explained. The bonding measure of friendship and the bridging measure of trust were most often associated with health measures. Linking social capital was the least likely component to be measured, although various studies found significant relationships with health outcomes. In the study of Veenstra [31] for example, political trust was a strong predictor of long-term illness and self-rated health. Hyyppä [32] found general mistrust to be correlated with negative health effects, but other social capital measures related to friendship and religious participation produced stronger effects. Although these findings confirm the co-existence of high social capital, high socioeconomic status and good health, they do not explain differences in this relationship between groups in society and might therefore obscure interaction effects.

### Buffer effect of social capital on health inequalities

The previous results identified a correlation between social capital and socioeconomic inequalities in health. Tables 1 and 2 show nineteen studies that sought to explain and nuance these findings by studying interaction effects. The buffer hypothesis suggests that people with a low socioeconomic status can use social capital as a buffer against the negative impact of low economic and/or cultural capital on health (Figure 1). There were eighteen studies that looked at the effect of socioeconomic status on the relationship between social capital and health, of which eleven confirmed the buffer hypothesis (Table 1).

**Table 1:** Studies reporting an interaction between social capital and socioeconomic inequalities in health

**Study (year)**	**Sample**	**Social capital measure**	**Health measure**	**Measure SES**	**Confirmed interaction hypothesis**	**Quality 1=poor, 2=average, 3=high**
**Abdou (2010)**	297 pregnant US women	Cognitive Bonding	Symptoms of mental illness, wellbeing	Childhood + adult SES	Buffer	2
**Altschuler (2004)**	49 Americans	Cognitive Structural Bridging	Self-rated health	Average household income	Buffer	2
**Baron-Epel (2008)**	4350 adult Jews and Arabs in Israel	Cognitive Structural Bonding	Self-rated health	Income, occupation and education	Buffer + dependency	2
**Beaudoin (2009)**	5586 US residents	Cognitive Structural Bridging	Self-rated health	Household income	Dependency	2
**Bohn (2011)**	4323 German students	Cognitive Structural Bonding Bridging	Self-rated health	Education	Buffer	3
**Cohen (2003)**	8782 Chicago residents	Cognitive Bridging	Premature mortality	Concentrated neighbourhood disadvantage	Buffer	2
**Gee (2006)**	2241 Filipino Americans	Cognitive Bonding	Unfair medical treatment	EducationEmployment	Buffer	3
**Gorman (2007)**	29816 US citizens ≥ 25 years old	Cognitive Structural Bonding Bridging	Self-rated health Hypertension	Education, relative family income, employment, financial barriers, insurance	Buffer + dependency	3
**Jesse (2006)**	130 low-income pregnant US women	Cognitive Bonding	Smoking and substance abuse	Level of education, insurance status	Buffer	2
**Pearson (2011)**	8566 Americans	Cognitive Structural Bridging Bonding	Self-rated health	Education, household income	Buffer	2
**Stafford (2008)**	9082 UK residents	Cognitive Structural Bridging Bonding	Common mental disorders	Household deprivation	Buffer	3
**Subramanian (2002)**	21456 US residents	Cognitive Bridging	Self-rated health	Educational attainment, income	Area-level	3
**Sun (2009)**	1605 Chinese urban residents ≥ 15 years old	Cognitive Structural Bonding Bridging	Self-rated health	Education, poverty, household income	Buffer	3
**Van der Wel (2007)**	11807 residents from Oslo (Norway)	Cognitive Structural Bridging	Self-rated health	Median income, income inequality, education	Buffer	2

**Table 2:** Studies falsifying an interaction between social capital and socioeconomic inequalities in health

**Study (year)**	**Sample**	**Social capital measure**	**Health measure**	**Measure SES**	**Rejected hypothesis**	**Quality 1=poor**, **2=average**, **3=high**
**Abdou (2010)**	297 pregnant US women	Cognitive Bonding	Symptoms of mental illness, wellbeing	Childhood + adult SES	Dependency	2
**Abel (2011)**	3068 Dutch and Hungarian adolescents	Cognitive Structural Bonding	Self-rated health	Self-assessed financial resources	Buffer + dependency	2
**Bjornstrom (2011)**	2176 Los Angeles residents	Cognitive Bridging	Self-rated health	Relative income	Buffer + dependency	3
**Dahl (2010)**	3190 Norwegian adults	Cognitive Structural Bonding Bridging Linking	Self-rated health Longstanding illness	Education, employment status, subjective poverty, household income	Buffer + dependency	2
**Engstrom (2008)**	31182 adults from Stockholm, Sweden	Cognitive Structural Bonding Bridging Linking	Self-rated health	Occupation, education, income, area income	Buffer + dependency	3
**Gallo (2006)**	304 San Diego residents	Cognitive Structural Bridging Linking	Self-rated health	Education	Buffer + dependency	2
**Sun (2009)**	1605 Chinese urban residents ≥ 15 years old	Cognitive Structural Bonding Bridging	Self-rated health	Education, poverty, household income	Dependency	3
**Van der Wel (2007)**	11807 residents from Oslo (Norway)	Cognitive Structural Bridging	Self-rated health	Median income, income inequality, education	Dependency	2

Studies that focussed on minority populations provided valuable insights, such as the research by Pearson and colleagues [33], who concluded that - especially for low-income American Jews - ties bonding according to religion were related to better self-rated health. In an underdeveloped area in Western China, Sun and colleagues [34] observed an association between self-rated health and social capital only for residents suffering from deprivation. Social capital was measured as individually assessed neighbourhood cohesion, reciprocity and social support. Van der Wel [35] studied the effect of trust and volunteering at a neighbourhood level among residents of Norwegian communities. Communities rich in social capital (measured by aggregating individual responses to social capital questions) were found to exhibit an impact of social capital that only benefited self-rated health of the lowest income group, while no effect on health could be observed for residents with a higher income. Stafford and colleagues [36] found a buffer effect for contact amongst local friends, but a negative effect for attachment to the neighbourhood on common mental disorders. A study from Germany developed a specific social capital index for eleven to fifteen year olds and reported the strongest effect of school and neighbourhood social capital on self-rated health for children with the lowest level of education [37].

Studies that did not find a buffer effect are presented in Table 2. They used a variety of social capital measures ranging from neighbourhood satisfaction to trust, civic participation and political participation. The authors of these studies discuss various explanations for their findings. In a study from Norway the absence of a buffer effect is attributed to the low level of income inequality in the society under study [38], whereas a study from Sweden did not show a significant effect when analysing contextual and individual social capital separately [39].

### Dependency effect of social capital and socioeconomic inequalities on health

In three out of nineteen papers reporting on interaction effects it is argued that there is a dependency between social capital and socioeconomic inequalities in health. Baron-Epel [40] found evidence for both hypotheses of interaction in one Israeli sample. For the Arab ethnic minority, in line with the buffer hypothesis, social support was positively correlated with health. For the more affluent Jewish group bridging and linking types of social capital were significantly associated with higher self-rated health as well. A large survey conducted in the United States found an interaction between education, the probability of hypertension and social integration measured as participation in six different activities [41]. Those who did not finish high school saw their probability of hypertension increased with more social integration, while social integration was protective of hypertension in all groups who had received more education. The same interaction effect was shown for the social capital indicator ‘visited friends or family’. Beaudoin [42] compared groups of White and Black Americans plus high and low income groups and concluded that self-rated health of high income Whites profited most from high social capital, while poor Black Americans profited least. Eight studies, of which key findings are presented in Table 2, rejected the dependency hypothesis. Some of these reported they found a buffer effect instead [34,35,43], while others confirmed a dependency effect only for certain populations [44] or rejected any type of interaction effect [38,39,45,46]. Bjornstrom [45], interestingly, did not find a relationship between health and relative position, although a significant relationship between health, family income and social capital was confirmed. Studies that could not identify a buffer or dependency relationship mostly used data from European countries, suggesting that the relationship between social capital and socioeconomic inequalities in health might differ across countries.

### Effect of contextual social capital on health inequality

Studies that aggregated individually measured data to an area level generally did not produce significant results [35,39]. Engstrom and colleagues [39] found an effect of contextual social capital on self-rated health, but this was no longer significant when adjusting for the effect of individual socioeconomic status. This finding indicates that initial results reflected the effects of individual socioeconomic status on health rather than the effect of contextual social capital. However, one large US study did show an effect for contextual bridging social capital on a community level [47]. This significant effect disappeared after controlling for individual factors in a multilevel analysis, but further analysis of subgroups showed an interaction with individual trust. For people who reported a high level of trust, community level trust was protective of health, while for people with a low trust score, high community level trust negatively affected health. This result reported by Subramanian [47] is in line with the dependency hypothesis of individual social capital, since both suggest a lack of social capital or inability to use it for people with a lower socioeconomic position. However, it remains unclear whether neighbourhood social capital truly is an attribute of the community or simply a reflection of individual social capital.

## Discussion

### Summary of key findings

This review provides an overview of current evidence on the associations between social capital and socioeconomic inequalities in health. Findings from a total of sixty studies can be summarised into four categories. Firstly, there is strong evidence to suggest that people with a lower socioeconomic status generally have lower levels of social capital, and that lack of social capital is related to socioeconomic inequalities in health. This hypothesis is supported by studies with various designs, sample sizes and settings [48-59]. The studies report on different types of social, economic and cultural capital, although the choice of a certain measure is not always based on a thorough theoretical framework.

Secondly, there is an indication that social capital, especially bonding social capital between close relations or tight-knit communities, can buffer some of the negative effects of low socioeconomic status on health [33-37,40,43,60-64]. Studies confirming this hypothesis generally focussed on social capital measured at the individual level and most significant buffer effects were observed among deprived communities and ethnic minorities. These findings are supported by literature on ethnic density, which suggests that ethnic minorities concentrated within neighbourhoods have better health outcomes than would be expected based on their, often low, socioeconomic position. Recently, two extensive literature reviews have shown some evidence of this effect for mortality, physical morbidity, health behaviour and mental health [65,66].

Thirdly we find that disadvantaged groups or people can be restricted in their opportunities to obtain and use social capital [40,42,63]. This hypothesis is consistent with the concept of social capital as described by Bourdieu. In much of his writing social capital is pictured as an asset of the privileged and a means of maintaining their superiority [67].

Our fourth hypothesis focuses on the negative effects of bridging and linking social capital for individuals with low economic capital [47]. Groups that do not have access to bridging social capital in a community might be better off in an environment where bridges between people are less strong, rather than in a community where disadvantaged groups are socially excluded. It has been shown before that poor mothers are less healthy in affluent areas compared to more deprived areas, suggesting an important role for psychosocial factors in the risk of illness [68].

### Strengths and limitations

This study is the first to systematically review the literature on the relationship between social capital and socioeconomic inequalities in health. We sought evidence for interaction effects between social capital, health and social inequality that have been discussed in previous research, but never before backed up by an overview of relevant studies on the subject. Whereas social capital has previously been taken for granted as a health benefit, our review distinguishes two main pathways that lead to a positive health effect for some and no or negative effects for others, depending on socioeconomic position.

Limitations should be taken into account when interpreting the results from this systematic review. Firstly, it is possible that our results are biased because relevant studies have not been identified through the literature search. However, apart from excluding grey literature our search was deliberately broad to include all definitions and measures of social capital and different interpretations of ‘socioeconomic inequalities in health’. To further reduce the risk of selection bias, all studies were screened by two researchers independently and reasons for disagreement were discussed. Aiming at maximum transparency of the selection process, we have reported all reasons for exclusion after initial disagreement (Additional file 2).

Secondly, findings of this review may be affected both by the quality of individual studies and by bias across studies. We rated the quality of individual studies with special emphasis on the suitability and validity of social and economic capital measures to clarify the relationship between social capital and socioeconomic inequalities in health. The quality of thirty-one out of sixty studies was rated suboptimal, mainly because they failed to address social capital based on a sound theoretical framework, resulting in a seemingly arbitrary choice of measurement (Additional file 3). Fortunately, the other half of the studies did base their research on a theoretical discussion of the social capital concept. Cene [69] for instance performed a qualitative study based on the framework developed by Carpiano [70] and others used standardised questionnaires for the measurement of social capital and related concepts. An example of the latter is the study by Johnson [71], which makes use of a social capital index consisting of six items with tested internal consistency.

A third limitation of the study, relating to the interpretation of findings, is that none of the hypotheses are confirmed by all included studies, and the finding that social capital can lead to social exclusion for people with a lower socioeconomic position is only supported by five studies out of nineteen. The majority of research does not specifically address the interaction between social capital and socioeconomic inequalities in health. However, studies generally made use of large samples, often representing a diverse population in terms of age, gender and ethnicity. Findings indicate a growing interest in this area since 2006. There is a shift from confirming and emphasizing the contribution of psychosocial factors to health inequalities, to a more in-depth study of these psychosocial pathways, in an attempt to explain the social gradient in health. Our study contributes to this trend, and hopefully more studies will follow with the aim to test the identified hypotheses.

### Implications for research and policy

This review once more confirms the correlation between social capital, socioeconomic inequalities and health. Evidence for the buffer hypothesis, the dependency theory and the area-level interaction however remains much weaker. Nevertheless, it is worth considering the implications of these theories.

Findings relevant to the buffer hypothesis have resulted in a call for the stimulation of social capital in vulnerable groups, such as the suggestion by Waterston [72] that social participation in neighbourhoods can protect children from the negative health effects of being poor and even has the power to decrease infant mortality. Putnam advocates a revival of social capital in American society, which he argues should be achieved primarily through civic engagement [9]. In 2010 the UK Conservative party launched their ‘Big society’ vision, based upon the idea that stimulating community participation and cohesion would empower people to bring positive changes to their communities from the bottom up. These and similar initiatives have received two major points of criticism. Firstly, recent literature suggests that the promotion of social capital through togetherness and social cohesion is based on the social norms and values of the empowered religious and ethnic majority, not taking into account minority groups that deviate from the norm [23]. Religious participation for example will only appeal to those who consider themselves religious, while interventions at work will exclude the unemployed. Sports clubs are a way to promote health and sociability, but their facilities might not be compatible with certain cultural norms, and women can experience barriers to participation [73]. Secondly, researchers have stressed that policy implications of social capital research should be treated cautiously, since the emphasis of health promotion on self-advocacy through social capital holds the danger of blaming the victim [74,75]. Indeed, the idea that disadvantaged groups are to be held accountable for their position in society is likely to stimulate distrust and social exclusion. This will further reduce bridging and linking social capital among the groups that need it most. Paradoxically, Putnam himself showed in an early study of Italian society that it is distrust that makes people turn inward to their family, explaining bonding social capital not as a luxury but as a necessity to which people are forced by the negative influences of bridging and linking social capital in an unequal society [76].

This criticism touches upon a second implication of our findings related to the dependency and area-level hypotheses, namely that social capital is not a function of free choice, but restricted by external factors at the community level. Abel [77] discusses these consequences of Bourdieu’s framework in the light of socioeconomic inequalities and power differences. Those that hold substantial power in a society are able to acquire social capital, either for personal use or for the benefit of their network. Economic capital can be converted into social capital and vice versa, and with the accumulation and transmission of capital within a network, outsiders cannot access it for the benefit of their health. This concept of social capital seems more relevant for bridging and linking than for bonding capital, and Bourdieu indeed considers bonding social resources such as social support to be distinct concepts [70]. In line with Bourdieu, Coburn [75] argues that, especially in unequal societies based on a neo-liberal model, bridging social capital is only freely available to the better-off. Social inequalities are, according to Coburn, a requirement for the viability of capitalism, so that decreased social trust and cohesion are inevitable. On a societal level, this hypothesis complements the finding that Western countries with a high level of income inequality score worse on many health outcomes and social indicators than more equal countries [78]. Consequently, in order to build social capital successfully social inequalities would have to be actively reduced. Uslaner [79] uses a similar argument when he argues that trust cannot simply be built. If trust and equality are related, because those who form a minority in terms of power have little reason to trust, then reducing social inequality is inevitably part of the solution. The state reinforces inequality or stimulates equality, hereby affecting social capital. Social capital can in turn affect equality, in a positive way by the creation of a more cohesive society and in a negative way by promoting social exclusion. Social capital should thus be built not only from the bottom up but also facilitated from the top down [80].

## Conclusion

This review builds on existing literature to highlight two separate interaction effects between social capital and socioeconomic inequalities in health. These have been observed to contribute to the psychosocial pathway of health inequalities. Firstly, types of bonding and bridging social capital such as social support, social cohesion in a neighbourhood, close friends and emotional support from family members can buffer some of the negative effects of poverty on health, and might decrease the vulnerability of people with a lower position on the social ladder. Secondly, certain types of social capital might only benefit the health of those who have access to them through their having sufficient economic capital and it may harm the health of those who are excluded from participation in the relevant networks. Measures of social capital found to confirm this hypothesis include social support, trust, social integration and neighbourhood safety [40,42,63]. As evidence is limited, no conclusions can be drawn on the types of social capital through which this mechanism operates.

The debate in relation to social capital and health inequalities sees some advocate the building of social capital for health benefits, while others put an emphasis on the negative effects of social capital that they consider inherent to unequal modern societies. As we have shown that the various components of social capital may have multiple effects on the health of people with different positions in society, future research should establish whether promoting social capital can improve health for all. If the dependency between social capital and health inequalities is confirmed in future research, this implies the urge for structural changes of society to tackle the psychosocial pathway of health inequalities.

## Competing interests

The authors, Noortje Uphoff, Kate Pickett, Báltica Cabieses, Neil Small and John Wright, declare that they have no competing interests.

## Authors’ contributions

NU coordinated the study, designed the protocol, summarized results and led the writing of the manuscript. KP assisted in design of the study and writing. BC contributed to development of the protocol, extraction and summary of results and writing. NS contributed to interpretation of results and writing the manuscript. JW assisted in the design and writing of the manuscript. All authors have read and approved the final manuscript.

## Supplementary Material

Additional file 1 Systematic literature search. Click here for file

Additional file 2 Reasons for exclusion after initial disagreement (N=37). Click here for file

Additional file 3 Summary table. Click here for file
