# Supplementary material for: A systematic review of the relationships between social capital and socioeconomic inequalities in health: a contribution to understanding the psychosocial pathway of health inequalities
Source: Int J Equity Health. 2013 Jul 19;12:54. doi: 10.1186/1475-9276-12-54 (PMC3726325; doi:10.1186/1475-9276-12-54)
Supplement: Additional file 1 — Systematic literature search. [file 1475-9276-12-54-S1.docx]

Appendix A. Systematic literature search
25 June 2012

Searched in MEDLINE, EMBASE, CINAHL, Cochrane.
No limits applied

MEDLINE (searched through PUBMED)

Mesh terms:
"Socioeconomic Factors"[Mesh]
"Health Status Disparities"[Mesh]
"Social Support"[Mesh]
"Community Networks"[Mesh]
"Social Isolation"[Mesh]
"Trust"[Mesh]

"health status disparities" OR "health inequality" OR "health inequity" OR "health inequalities" OR "health disparity" OR "health disparities" OR "Health Status Disparities"[Mesh]

AND

"socioeconomic status" OR "social class" OR poverty OR poor OR income OR disadvantaged OR deprivation OR deprived OR "socioeconomic factors" OR "socioeconomic position" OR deprivation OR deprived OR "Socioeconomic Factors"[Mesh]

AND

"social capital" OR "social support" OR "social participation" OR trust OR "emotional support" OR "social network" OR "social cohesion" OR "psychosocial support" OR "community capital" OR "neighbourhood cohesion" OR "neighborhood cohesion" OR "collective efficacy" OR "Social Support"[Mesh] OR "Community Networks"[Mesh] OR "Social Isolation"[Mesh] OR "Trust"[Mesh]

**N = 413**

EMBASE (searched through Ovid)

EMBASE subject headings (Emtree):
health disparity/
social status/
poverty/
social class/
social capital/
social support/
social isolation/
social network/
social participation/

"health status disparities" OR "health inequality" OR "health inequity" OR "health inequalities" OR "health disparity" OR "health disparities" OR health disparity/

AND

"socioeconomic status" OR "social class" OR poverty OR poor OR income OR disadvantaged OR deprivation OR deprived OR "socioeconomic factors" OR "socioeconomic position" OR deprivation OR deprived OR social status/ OR poverty/ OR social class/

AND

"social capital" OR "social support" OR "social participation" OR trust OR "emotional support" OR "social network" OR "social cohesion" OR "psychosocial support" OR "community capital" OR "neighbourhood cohesion" OR "neighborhood cohesion" OR "collective efficacy" OR social capital/ OR social support/ OR social isolation/ OR social network/ OR social participation/


**N = 355**

CINAHL

CINAHL subject headings:
(MH "Socioeconomic Factors") 
(MM "Health Status Disparities")
(MH "Social Capital")
(MH "Social Networks")
(MH "Social Isolation")
(MH "Trust")

"health status disparities" OR "health inequality" OR "health inequity" OR "health inequalities" OR "health disparity" OR "health disparities" OR (MM "Health Status Disparities")

AND

"socioeconomic status" OR "social class" OR poverty OR poor OR income OR disadvantaged OR deprivation OR deprived OR "socioeconomic factors" OR "socioeconomic position" OR deprivation OR deprived OR (MH "Socioeconomic Factors")

AND

"social capital" OR "social support" OR "social participation" OR trust OR "emotional support" OR "social network" OR "social cohesion" OR "psychosocial support" OR "community capital" OR "neighbourhood cohesion" OR "neighborhood cohesion" OR "collective efficacy" OR
(MH "Social Capital") OR (MH "Social Networks") OR (MH "Social Isolation") OR (MH "Trust")

**N = 92**

Cochrane

"health status disparities" OR "health inequality" OR "health inequity" OR "health inequalities" OR "health disparity" OR "health disparities" OR "Health Status Disparities"[Mesh]

AND

"socioeconomic status" OR "social class" OR poverty OR poor OR income OR disadvantaged OR deprivation OR deprived OR "socioeconomic factors" OR "socioeconomic position" OR deprivation OR deprived OR "Socioeconomic Factors"[Mesh]

AND

"social capital" OR "social support" OR "social participation" OR trust OR "emotional support" OR "social network" OR "social cohesion" OR "psychosocial support" OR "community capital" OR "neighbourhood cohesion" OR "neighborhood cohesion" OR "collective efficacy" OR "Social Support"[Mesh] OR "Community Networks"[Mesh] OR "Social Isolation"[Mesh] OR "Trust"[Mesh]

**N = 1**
