# Supplementary material for: A systematic review of the relationships between social capital and socioeconomic inequalities in health: a contribution to understanding the psychosocial pathway of health inequalities
Source: Int J Equity Health. 2013 Jul 19;12:54. doi: 10.1186/1475-9276-12-54 (PMC3726325; doi:10.1186/1475-9276-12-54)
Supplement: Additional file 2 — Reasons for exclusion after initial disagreement (N=37). [file 1475-9276-12-54-S2.docx]

Appendix C. Reasons for exclusion after initial disagreement (N=37)

| **Paper** | **Reason for exclusion** |
| --- | --- |
| Ashing-Giwa, K. T., J. S. Tejero, et al. (2009). Cervical cancer survivorship in a population based sample. *Gynecol Oncol* 112(2): 358-364. | Ethnic inequalities instead of socioeconomic inequalities in health |
| Bardach, S. H., Y. N. Tarasenko, et al. (2011). The role of social support in multiple morbidity: self-management among rural residents. *J Health Care Poor Underserved* 22(3): 756-771. | Rural/ spatial inequalities instead of socioeconomic inequalities in health |
| Coday, M., L. M. Klesges, et al. (2002). Health Opportunities with Physical Exercise (HOPE): social contextual interventions to reduce sedentary behavior in urban settings. *Health Educ Res* 17(5): 637-647. | No socioeconomic inequalities |
| Dailey, D. E. (2006). Social stressors and personal resources associated with perinatal outcomes among African American women, University of California, San Francisco. Ph.D.: 163 p. | Grey literature |
| Dong, X., M. Simon, et al. (2010). A cross-sectional population-based study of elder self-neglect and psychological, health, and social factors in a biracial community. *Aging Ment Health* 14(1): 74-84. | No socioeconomic inequalities |
| Emmons, K. M., E. M. Barbeau, et al. (2007). Social influences, social context, and health behaviors among working-class, multi-ethnic adults. *Health Education & Behavior* 34(2): 315-334. | No socioeconomic inequalities |
| Finlayson, T. L., K. Siefert, et al. (2007). Psychosocial factors and early childhood caries among low-income African-American children in Detroit. *Community Dent Oral Epidemiol* 35(6): 439-448. | No socioeconomic inequalities |
| Hodge, D. R., F. F. Marsiglia, et al. (2011). Religion and Substance Use among Youths of Mexican Heritage: A Social Capital Perspective. *Social Work Research* 35(3): 137-146. | No socioeconomic inequalities |
| Holden, C., N. Moses, et al. (2011). Collaborating to address infant mortality: lessons learned from the Brownsville action community for health equality. *Prog Community Health Partnersh* 5(3): 281-288. | Does not discuss the relationship between health and social capital |
| Murdock, K. K., E. M. Robinson, et al. (2009). Family-school connections and internalizing problems among children living with asthma in urban, low-income neighborhoods. *J Child Health Care* 13(3): 275-294. | No socioeconomic inequalities |
| Schulz, A. J., S. Kannan, et al. (2005). Social and physical environments and disparities in risk for cardiovascular disease: the healthy environments partnership conceptual model. *Environmental Health Perspectives* 113(12): 1817-1825. | A protocol for research; no research findings presented yet |
| Song, L. and N. Lin (2009). Social capital and health inequality: evidence from Taiwan. *J Health Soc Behav* 50(2): 149-163. | No socioeconomic inequalities |
| Zoellner, J. M., C. C. Connell, et al. (2011). H.U.B city steps: methods and early findings from a community-based participatory research trial to reduce blood pressure among African Americans. *Int J Behav Nutr Phys Act* 8: 59. | No socioeconomic inequalities |
| Bell, C. N., R. J. Thorpe, Jr., et al. (2010). Race/Ethnicity and hypertension: the role of social support. *Am J Hypertens* 23(5): 534-540. | Ethnic inequalities instead of socioeconomic inequalities in health |
| Campbell, C. and C. McLean (2002). Ethnic identities, social capital and health inequalities: factors shaping African-Caribbean participation in local community networks in the UK. *Soc Sci Med* 55(4): 643-657. | Ethnic inequalities instead of socioeconomic inequalities in health |
| Campbell, C. and C. McLean (2003). Social capital, local community participation and the construction of pakistani identities in England: implications for health inequalities policies. *J Health Psychol* 8(2): 247-262. | Ethnic inequalities instead of socioeconomic inequalities in health |
| Cislo, A. M., N. J. Spence, et al. (2010). The mental health and psychosocial adjustment of Cuban immigrants in south Florida. *Soc Sci Med* 71(6): 1173-1181. | Ethnic inequalities instead of socioeconomic inequalities in health |
| Jackson, M. N., M. A. Meade, et al. Perspectives on networking, cultural values, and skills among African American men with spinal cord injury: A reconsideration of social capital theory. *Journal of Vocational Rehabilitation*(of Publication: 2006): 25 (21) (pp 21-33), 2006. | Does not discuss the relationship between health and social capital |
| Jaksic, Z. Social determinants and epidemiology of cardiovascular diseases. [Croatian]. *Acta Medica Croatica*(of Publication: 2007): 61 (63) (pp 319-327), 2007. | Very broad discussion of social determinants; not specified to social capital, health and socioeconomic inequalities |
| McLean, C., C. Campbell, et al. (2003). African-Caribbean interactions with mental health services in the UK: experiences and expectations of exclusion as (re)productive of health inequalities. *Soc Sci Med* 56(3): 657-669. | Ethnic inequalities instead of socioeconomic inequalities in health |
| Rachlis, B. S., E. J. Mills, et al. Livelihood security and adherence to antiretroviral therapy in low and middle income settings: A systematic review. *PLoS One* 6 (5) , 2011. Article Number(of Publication: 2011): e18948. | Does not address (components of) social capital |
| Van Duyn, M. A., T. McCrae, et al. (2007). Adapting evidence-based strategies to increase physical activity among African Americans, Hispanics, Hmong, and Native Hawaiians: a social marketing approach. *Prev Chronic Dis* 4(4): A102. | Ethnic inequalities instead of socioeconomic inequalities in health |
| Chandola, T. (2012). Spatial and social determinants of urban health in low-, middle- and high-income countries. *Public health.* 126: 259-261. | symposium report |
| Cohen, D.A., Inagami, S., Finch, B. (2008). *Health & Place*. 14: 198-208. | no health measure included |
| Elovainio, M., J. E. Ferrie, et al. (2011). Socioeconomic differences in cardiometabolic factors: social causation or health-related selection? Evidence from the Whitehall II Cohort Study, 1991-2004. *Am J Epidemiol* 174(7): 779-789. | no analysis of social capital |
| Kramer, M.R., Cooper, H.L., Drews-Botsch, C.D. et al. (2010) Metropolitan isolation segregation and Black-White disparities in very preterm birth: A test of mediating pathways and variance explained. 71: 2108-2116. | no analysis of social capital |
| Litaker, D., S. M. Koroukian, et al. (2005). Context and healthcare access: looking beyond the individual. *Med Care* 43(6): 531-540. | no analysis of social capital |
| Mackerth, C., Appleton, J. (2008). Social networks and health inequalities: evidence for working with disadvantaged groups. *Community Practitioner*. 81(8):23-6. | no quantitative or qualitative analysis |
| Novak, M., Ahlgren, C., Hammarstrom, A. (2012) Social and health-related correlates of intergenerational and intragenerational social mobility among Swedish men and women.*Public Health* 126: 349-357. | no analysis of social capital |
| Roberts, E.M. (1997) Neighborhood social environments and the distribution of low birthweight in Chicago. *Am J Public Health*. 87:597-603. | no analysis of social capital |
| Gonzalez-Perez, G. J., M. G. Vega-Lopez, et al. (2008). [A socio-spatial analysis of social exclusion and inequity in health in Mexico]. *Rev Salud Publica* (Bogota) 10 Suppl: 15-28. | no analysis of social capital |
| Gonzalez-Perez, G. J., M. G. Vega-Lopez, et al. (2011). [Demographic characteristics, social inequality and inequity in Mexican childhood health]. *Rev Salud Publica* (Bogota) 13(1): 41-53. | no analysis of social capital |
| Dixon, E. L. (2004). Neighborhoods and adult health status: A multi-level analysis of social determinants of health disparities in Los Angeles County, University of California, Los Angeles. Ph.D.: 153 p. | grey literature |
| Wallerstein, N., R. Mendes, et al. (2011). Reclaiming the social in community movements: perspectives from the USA and Brazil/South America: 25 years after Ottawa. Health Promot Int 26 Suppl 2: ii226-236. | no analysis of social capital |
| Hajna, S., N. Ross, et al. The role of neighborhood-level social and material deprivation on walking in adults with type 2 diabetes. Diabetes Conference(Start: 20110624 Conference End: 20110628): 71st Scientific Sessions of the American Diabetes Association San Diego, CA United States. | grey literature |
| Ebrahim, S. H., J. E. Anderson, et al. (2009). Overcoming social and health inequalities among U.S. women of reproductive age -- challenges to the nation's health in the 21st century. *Health Policy* 90(2-3): 196-205. | no analysis of social capital |
| Marmot, M. G., G. D. Smith, et al. (1991). Health inequalities among British civil servants: the Whitehall II study. *Lancet*. 337(8754): 1387-1393. | background information; no analysis of social capital |
